# Feeding efficiency gains can increase the greenhouse gas mitigation potential of the Tanzanian dairy sector

**DOI:** 10.1038/s41598-021-83475-8

**Published:** 2021-02-18

**Authors:** James Hawkins, Gabriel Yesuf, Mink Zijlstra, George C. Schoneveld, Mariana C. Rufino

**Affiliations:** 1 Lancaster Environment Centre, Lancaster University, Lancaster, UK; 2 Plant Production Systems, Wageningen University, Wageningen, The Netherlands; 3 Center for International Forestry Research (CIFOR), Bogor, Indonesia

**Keywords:** Climate-change mitigation, Environmental impact

## Abstract

We use an attributional life cycle assessment (LCA) and simulation modelling to assess the effect of improved feeding practices and increased yields of feed crops on milk productivity and GHG emissions from the dairy sector of Tanzania’s southern highlands region. We calculated direct non-CO_2_ emissions from dairy production and the CO_2_ emissions resulting from the demand for croplands and grasslands using a land footprint indicator. Baseline GHG emissions intensities ranged between 19.8 and 27.8 and 5.8–5.9 kg CO_2_eq kg^−1^ fat and protein corrected milk for the Traditional (local cattle) and Modern (improved cattle) sectors. Land use change contributed 45.8–65.8% of the total carbon footprint of dairy. Better feeding increased milk yields by up to 60.1% and reduced emissions intensities by up to 52.4 and 38.0% for the Traditional and Modern sectors, respectively. Avoided land use change was the predominant cause of reductions in GHG emissions under all the scenarios. Reducing yield gaps of concentrate feed crops lowered emissions further by 11.4–34.9% despite increasing N_2_O and CO_2_ emissions from soils management and input use. This study demonstrates that feed intensification has potential to increase LUC emissions from dairy production, but that fertilizer-dependent yield gains can offset this increase in emissions through avoided emissions from land use change.

## Introduction

Tanzania is a low-income country of East Africa characterized by relatively low agricultural productivity and a national greenhouse gas (GHG) emissions profile dominated by the land use sector. Land use change (LUC) is the largest contributor to national GHG emissions, representing 66.0% of its estimated 319 Mt of annual CO_2_eq emissions, with agricultural emissions (excluding LUC) accounting for 18.8% of these emissions^1^. About 55% of Tanzania’s land area is occupied by woodlands and forests, and these areas are under increasing pressure from anthropogenic activities, especially agriculture^2^. The expansion of land areas for crops and grazing are the two largest causes of deforestation in the country^3^. The country has committed to reduce emissions by 10–20% relative to the business as usual scenario by 2030 under the Paris Agreement^4^, although to date, the agricultural sector is not included in Tanzania’s nationally determined contribution (NDC). The implementation of climate change mitigation initiatives in the land and agriculture sectors is hampered by conflicts with economic development objectives^5^ and by the lack of foresight analyses linking the impact of proposed GHG mitigation strategies to changes in emissions and productivity^6^.

In the coming years, growth in demand for milk and dairy products caused by rising urban consumption is expected to lead to a national milk supply gap of 5600 Mg year^−1^ by 2030^7^. The Tanzanian Livestock Master Plan (hereafter LMP) is a development program that, amongst others, aims to close this milk supply gap in order to alleviate poverty and raise rural incomes^8^. There is potential for concurrently including Tanzania’s dairy sector in the NDC and the development initiatives in the LMP; this, because the LMP prioritizes productivity growth as a means to closing the projected supply gap. Such measures, via their effect on improving feed conversion efficiency, could result in reductions in GHG emissions intensities (Herrero et al.^9^), potentially producing win–win outcomes should these two initiatives be combined. To increase the likelihood of success of these mitigation policy initiatives, a framework is required for quantifying the GHG emissions reductions possible in reference to a baseline^10^, for which no such analysis has been done.

From a practice point of view, better livestock diets are widely viewed as essential to improving productivity and reducing GHG emissions from dairy^11^. Tanzania’s dairy sector is constrained by lack of adequate feed resources, associated with a widespread degradation of grasslands, land shortages in some regions, poor uptake of better forage production and conservation practices, and a poorly developed animal feed processing industry^8,12^. Such factors lead to significant seasonal variations in milk production and offtake^13^. Dry season feed deficits and the low genetic potential of much of the herd limits milk productivity growth, and lead to a high national average emissions intensity of 19.9 kg CO_2_eq kg^−1^ FPCM (fat and protein corrected milk)^14^. Kenya and Ethiopia emit 3.8 and 24.5 kg CO_2_eq kg^−1^ FPCM, respectively^15,16^, indicating that there is room for improvement. Feeding management can influence productivity and GHG emissions in multiple ways. Adding more nutrient-dense feeds to diets can improve milk yields and reduce methane (CH_4_) emissions intensity^17^. However, higher total energy content of diets can also increase methane production per animal^18^. Other risks include increasing CO_2_ emissions from expanding cropland areas^19^ and N_2_O emissions from intensification of feed crop production^19^. Changes in feeding practices can also lead to land sparing by substituting low yielding grass and forages with higher yielding feed crops, for which regional and global studies have suggested can reduce grassland requirements^20^ and reduce deforestation^21^. As an estimated 96% of cattle in Tanzania are reared in extensive grazing systems^22^, we hypothesized that land sparing is a leading strategy for reducing dairy GHG emissions.

This study assessed the effect of improved feed management in Tanzania’s dairy sector on GHG emissions in relation to the output growth targets of the LMP. The analysis sought evidence for the merits of linking the LMP to climate change mitigation initiatives, such as a dairy sector Nationally Appropriate Mitigation Action (NAMA). We used a life cycle assessment (LCA) to quantify GHG emissions adding on previous work by Mottet et al.^23^, Brandt et al.^24,25^ and Notenbaert et al.^26^. While all these studies accounted for the role of improved productivity in reducing direct dairy sector emissions, to date no study has evaluated specifically the role of land sparing and the potential for avoided land use change emissions to contribute to reductions in the dairy carbon footprint for Tanzania’s dairy. For this purpose, we employed a land footprint indicator, which has been used previously for assessing GHG emissions and productivity indicators of ruminant livestock systems in sub-Saharan Africa^27,28^. This indicator helps assess the implications of crop and grassland expansion on LUC emissions and is consistent with the IPCC Guidelines for National Greenhouse Gas Inventories^29,30^. The objective was to quantify the impact of improved feeding management on milk output and sectoral emissions by 2030. The study focussed on high-productivity systems of the southern highlands regions of Njombe, Mbeya, and Iringa and the Morogoro region. This region is well suited agro-ecologically for dairy production, and is increasingly attracting private and public sector investments in order to secure milk production for growing urban centres such as Mbeya and Dar es Salaam^31^.

## Methods

### Modelling approach and data sources

**Figure 1:**
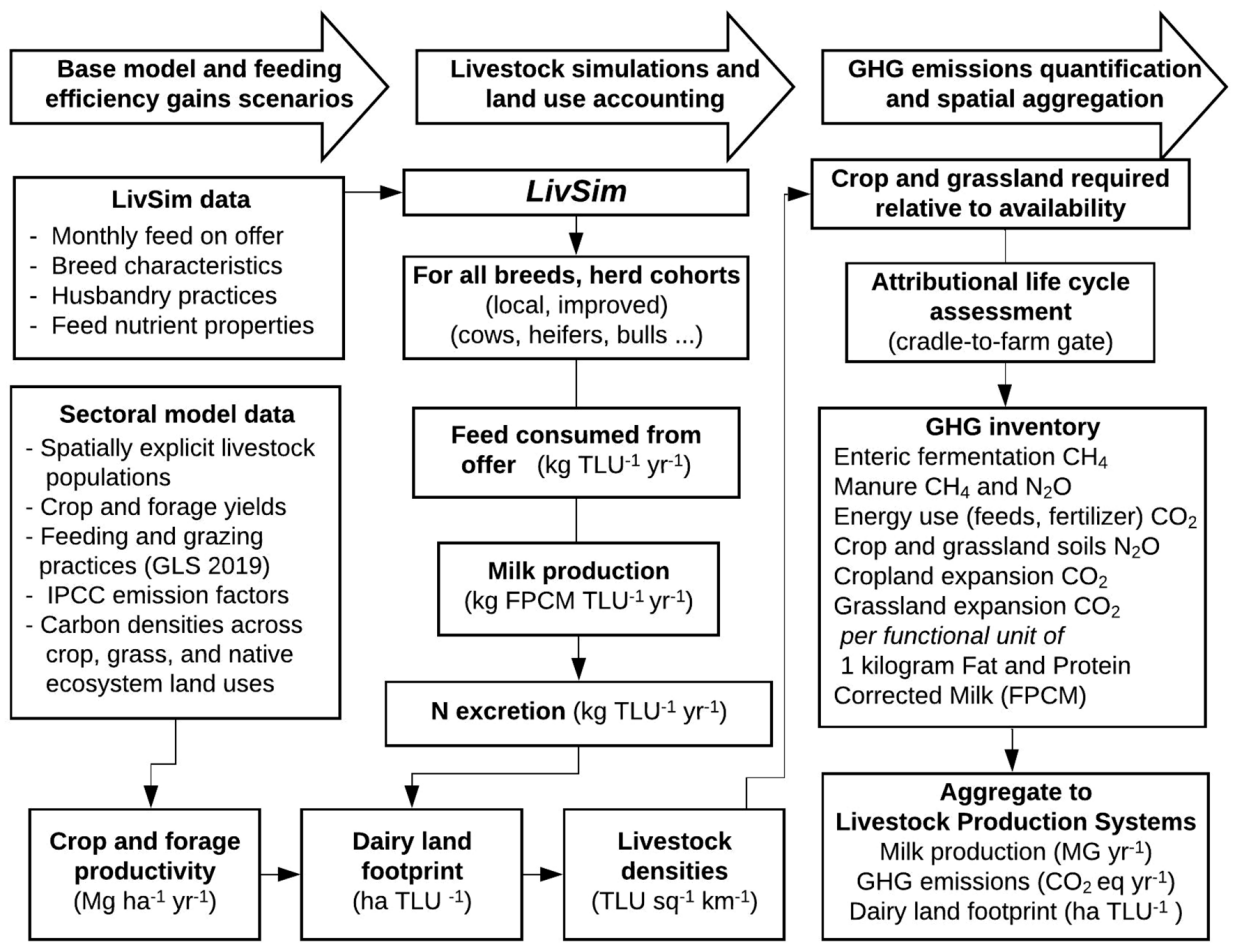
Analytical framework. A dynamic livestock simulation model (LivSim) is linked to an attributional life cycle assessment (LCA) and a spatial aggregation procedure to quantify GHG emissions per kilogram of fat and protein corrected milk (FPCM).

The analytical framework involves coupling the *Liv*estock *Sim*ulation model (*LivSim*) (Rufino et al.^32^), an algorithm to calculate the land footprint of the dairy sector, and a greenhouse gas quantification protocol based on principles of life cycle assessment (Fig. 1). *LivSim* is a dynamic model that simulates the lifetime productivity of dairy cows based on feeding and genetic potential^32,33^. LivSim was used to simulate individual cohorts of dairy animals (cows, bulls, juvenile males, heifers, calves) across their lifetime, and the milk production and GHG emission estimates are aggregated to the production system level. These form the basis for defining a baseline of milk production, emissions, and land use, and for assessing the impact of feeding efficiency gains. The model was coded in the Python programming language^34^ as a shell program that runs *LivSim* (also coded in Python) with additional code to define the land footprint and conduct the LCA (Fig. S1).

The land footprint indicator includes all land directly used for providing feed biomass: cultivated and grazing land, and land use ‘upstream’ from the farm for production of concentrate feeds. This framework allows an assessment of the impact of changes in diets, or in productivity gains through higher crop yields, to the changes in land use and milk productivity. The dairy land footprint, expressed as hectares per tropical livestock unit (250 kg liveweight), is as forth defined as all crop and grassland directly used for feeding dairy cattle:

1 $${\mathrm{Dairy \;land\; footprint}}_{\mathrm{b},\mathrm{s}}\left(\mathrm{ha \;}{\mathrm{TLU}}^{-1}\right)= {\sum }_{c=1}^{C}{\sum }_{f=1}^{F}\frac{{\mathrm{Feed \;on\; offer }}_{\mathrm{s},\mathrm{b},\mathrm{c},\mathrm{f}} }{{\mathrm{Yield }}_{\mathrm{f}} \times {\mathrm{ Use \;efficiency }}_{\mathrm{f}}}$$

where *b* represents the cattle breeds, *s* represents the livestock production systems, *C* represents the cattle cohorts, *F* represents the feeds included in the model, *Feed on offer* is the annual feed provision per TLU for a given breed, cohort and for a specific feed (Mg TLU^−1^ year^−1^), *Yield* the annual yield of the given feed (Mg ha^−1^ year^−1^), and *Use efficiency* the fraction of biomass that is either harvested or grazed. Feed on offer includes all feed available from grazing, harvested on-farm, or purchased from the market.

**Figure 2:**
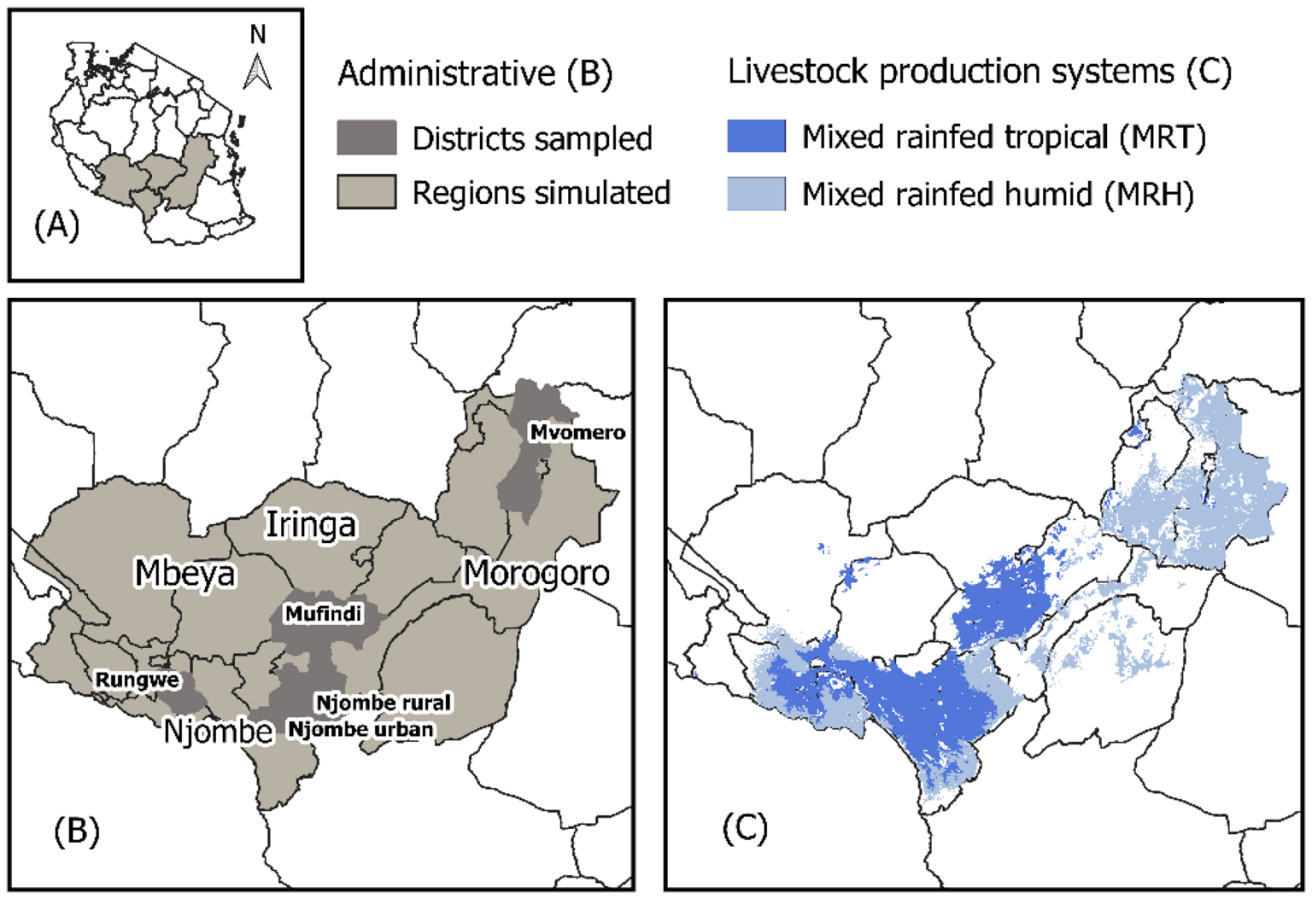
Geographic focus of study. (**A**) Shows the region within which the study focusses. (**B**) Shows the administrative regions (Mbeya, Njombe, Iringa, Morogoro) for which the model simulations were run and the districts (Rungwe, Njombe urban and rural, Mufindi, and Mvomero) the survey sampled from. (**C**) Shows the livestock production systems within which the simulations were conducted. Figure generated in QGIS 3.8.2^36^ (https://www.qgis.org/en/site/).

The model was parameterized with data from a survey of 1199 smallholder dairy farms conducted in southern Tanzania from November 2017 to August 2018. Surveying activities, performed as part of the IFAD-funded Greening Livestock project, were informed by a stratified random sampling protocol, capturing diversity in dairy farming households (by cattle breed, and socioeconomic factors) among mid to high potential systems across four sampled districts (Fig. 2). Baseline indicators characterizing existing feeding practices were developed, which in turn represent diets within the livestock simulations. For the remainder of this paper this survey dataset will be referred to as GLS^35^.

### Livestock systems and milk production in south and eastern Tanzania

This study focussed on mixed (M) crop-livestock production, rainfed (R), tropical (T) humid (H) systems (hereafter MRT, MRH), following the Robinson et al.^37^ classification. MRT and MRH systems comprise a total of 43,400 km^2^ (18,500 km^2^ MRT; 24,900 km^2^ MRH) across the four regions. In these regions, rainfall is unimodal; the rainy season stretches from November to April, followed by a 6-month dry period^38^. Feed sources within these systems depend, to varying degrees, on biomass consumed from grazing, crop residues, cultivated forages, and concentrates acquired off farm. Seasonal variation in feed quantity and quality leads to different grazing and feeding practices across seasons. During the dry season residues from crops form a larger percentage of diets due to the lower availability of natural and planted forages. Concentrates are available from the market year-round but they are generally used sparingly to improve productivity of cows and to maintain nutrient availability during periods of feed scarcity^39^. Protein-dense concentrates, especially sunflower cake, are used to improve milk yields of cows, while maize bran is commonly used as a supplement to maintain energy availability throughout the year^22^. Both of these feeds are produced and processed locally^22,40^. The baseline diets in the present study, including the seasonal biomass intake from cut-and-carry feeding systems, market purchases, and grazing, were specified using GLS^35^ and supplementary data sources^41,42^ (described in SI 1). Feed nutrient properties used in livestock simulations were derived from FAO^43^ and literature^44–46^ (Table S4) 

The land footprint was disaggregated based on the dominant sources of feed biomass, and the corresponding land uses (Table 1). This allows the impact of changes in croplands and grasslands to land use change emissions to be linked, as per the IPCC^29^ Guidelines. The main feed categories used were: primary crop products (sunflower cake and maize bran), secondary crop products (maize stover), and grass. Grasslands are further divided into native (unmanaged) and sown (managed). The nutritional value and biomass yield of native grasslands were based on the literature on predominant native grass species in the region. Two types of grasses were distinguished based on their yields and nutrient contents: low quality species of grasses were referred to as ‘Pasture’, which are either harvested or grazed, while ‘Napier grass’ (*Pennisetum purpureum*), which is the most common improved forage produced in the region (GLS^35^), is considered a high quality, high yielding forage used primarily in cut-and-carry systems.

**Table 1:** Biomass productivity, nitrous oxide fluxes, and carbon density parameters for feed and land use categories in model.

Land use	Feed	Annual yield	Available feed biomass	Use efficiency	Nitrous oxide flux	Carbon density		
		Mg DM ha^−1^	Mg DM ha^−1^ year^−1^	Fraction	kg N_2_O ha^−1^ year^−1^	Mg C ha^−1^		
						Soils^b^	Other pools^e^	Total
Croplands	Maize	1.46^d^	0.44 (bran) 2.18 (stover)	0.95	0.73 (stover) 1.03 (bran)	38.0	3.5	41.5
	Sunflower	1.03^d^	0.36 (cake)	0.95	0.90			
Grasslands	Napier grass	13.04^a^	13.10	0.75	0.51	48.0	4.5	52.5
	Pastures	10.00^c^	3.04	0.50	0.08			
	Grasslands	3.00^c^	1.50	0.50	0.13			
Wetlands						42.0	4.4	46.4
Shrubland						41.0	16.6	57.6
Forest						69.0	37.8	106.8

Sources: a^47^, b^48^, c^7^, d^40^, e^49^.

The fraction of feed available from the total biomass yield, which takes into account the use efficiency, harvesting and manufacturing ratios (e.g. the ratio of bran or cake obtained from the grain or seed portion of the crop) are shown in Table 1. The biomass available from crop residues was calculated using a harvest index of 0.35^50^. For concentrates the ratio of processed feed products (bran from maize or cake from sunflower) were obtained from literature^51,52^. The use efficiency ranges from 0.50 to 0.95, and were set to 0.50 for grass and pasture, consistent with values that have been used in previous assessments such as^53^. These values reflect the high stocking rates among highland grazing systems in Tanzania^54^, which result in 0.39–0.61 forage use efficiency^55^. The use efficiency for Napier grass was set at 0.75 based on harvesting ratios reported from field experimental trials in sub-Saharan Africa^56^. The use efficiencies for maize and sunflower were set at 0.95 which are consistent with the nationally reported harvesting efficiency of FAO Stat^40^. The feed biomass yields per feed type, land use classifications, baseline soil N_2_O fluxes (see “SI” for how these were estimated) and C densities of these land use types are shown in Table 1.

#### Dairy cattle populations and milk production

The dairy sector included all milking cows, replacement females (heifers and female calves), and reproductive cohorts (bulls, juvenile males, and male calves) which are required for maintaining the stock of cows. Between 90 and 98% of the cows milked in the study areas were indigenous (*Bos indicus*) cattle, while the other 2–10% were crossbred (*Bos indicus* × *Bos taurus*) or purebred (*Bos taurus*)^57,58^. Studies indicate that milk production by improved dairy cattle breeds ranges from 1350 to 2200 L lactation^−1^^57,59^ and calving intervals range from 400 to 520 days^59,60^. For indigenous cattle, milk yields are typically 500–600 L lactation^−1^, and calving intervals range from 450 to 600 days^59,60^. Due to the difference in productivity between local indigenous and improved cattle, this study disaggregated the dairy sector (and the dairy land footprint) by breed, resulting in two sectors: the Traditional (local cattle) and Modern (improved cattle) sectors. Livestock simulations for cattle in the respective sectors were conducted with breed parameters derived from literature^61–68^ (Table S1).

### Quantification of greenhouse gas emissions

The dairy sector’s GHG emissions were calculated using an attributional life cycle assessment^69^. The LCA boundary was defined as ‘cradle to farm gate’; all major GHG emissions sources from resource extraction through to the farm gate were included. Post-farm gate emissions such as for transporting and processing raw milk were not considered. Emissions sources were expressed in relation to a functional unit of one kilogram of fat and protein corrected milk (FPCM) which is calculated as milk production standardized to 4% fat and 3.3% protein (IDF)^70^. The inventory of GHG emissions sources (Fig. 1) included enteric fermentation (CH_4_), manure (CH_4_ and N_2_O), organic and inorganic N inputs into crop and grassland soils (N_2_O), energy use from manufacturing and transport of feed and fertilizer inputs (CO_2_), and land use change emissions (CO_2_) from changes in crop and grasslands driven by the direct changes resulting from increased demand from dairy cattle. A mass allocation factor was used to allocate the total GHG emissions from the dairy herd to production of milk and meat, and this value ranged from 0.85 to 0.95. Meat production was calculated using culling rates for each sex (7.7 and 14.0% for female and male cattle, respectively) and a dressing percentage of 52%^57,71^. Methane and nitrous oxide were converted to CO_2_ equivalents using global warming potentials of 28 kg CO_2_eq kg^−1^ of CH_4_ and 265 kg CO_2_eq kg^−1^ of N_2_O^72^. The GHG emissions from enteric fermentation, manure, and soils were calculated in line with IPCC^29^ guidelines taking emission factors derived from literature^73–75^ or estimated using equations from literature^76^ (SI 2). In cases where local emission factor data were not available, default IPCC (Tier 1) values were used. CO_2_ emissions from energy used during the manufacturing of fertilizer inputs, feed processing, and the transportation of feed and fertilizer to the farm were included by linking fertilizer and concentrate feed use to CO_2_ emissions using embodied emission factors obtained from the literature^77–80^ (SI 2). Sources of GHG emissions omitted include those from cattle respiration, farm machinery, electricity, inputs other than feeds and fertilizers, and the construction of farm structures, as these are generally considered minor especially in a low-income context^53^. The results of the baseline values of N_2_O fluxes modelled from IPCC equations (SI 2) from crop and grassland soils are shown in Table 1.

#### Carbon dioxide emissions from land use change

Land use changes attributed to changes in feed demand were categorized into one of two transitions: (1) *cropland expansion*: grasslands being converted to croplands, and (2) *grassland expansion:* other native ecosystems being converted to grasslands. Native ecosystems in this context included wetlands, shrubland, and forests. Indirect land use change from feed crops replacing grasslands is accounted for via the ‘competition effect’^81^. As croplands displace grasslands, a proportional increase in grassland expansion must take place to meet forage requirements. Thus, because *grassland expansion* can result in native ecosystems being displaced, *cropland expansion* (via the displacement of grasslands) can also indirectly lead to the conversion of native ecosystems.

The CO_2_ emissions from these land use changes were estimated using the stock change method^29,82^. Under this framework, the flux of C (Mg C ha^1^ year^−1^) resulting from the conversion of land is related to the difference in C densities between the current and the previous land use. The C densities for a given land use category are equal to the sum of the five following pools: soils, below and above ground biomass, coarse woody debris, and litter^29^. Following the practice of LUC accounting in dairy LCA, the CO_2_ emissions after land use change were amortized over a 20-year period^71,83^. The transition coefficient for *cropland expansion* was based on the differences between grassland and cropland C stocks reported in Table 1. This resulted in a difference of 11.0 ± 2.0 Mg C ha^−1^ between crop and grasslands.

#### Estimating CO_2_ emissions from conversion of native ecosystems to grasslands

The extent of grassland expansion was calculated based on the relative availability and utilization of grassland for both LPS based on the density of dairy cattle and availability of grassland per grid cell (see “SI” for details), following an approach similar to that of Havlik et al.^84^. Thus, native ecosystems were converted to grasslands when the demand for grasslands exceeded availability. To calculate the transition coefficient, native ecosystem C stocks were estimated using spatially-explicit land cover data at a 100 × 100 m pixel resolution^85^. The C stock density of native ecosystems was estimated as a weighted mean of the shrub, forest, and wetland categories. The C densities of these land categories (for the non-soil C pools) were based on national carbon stock inventory data^49^ and for soils, based on a topsoil dataset compiled from 1400 locations across Tanzania^48^ (Table 1). The weights were based on the proportion of shrub, forest, and wetland in a given grid cell^85^. This data was up-scaled to the same spatial resolution as the LPS data and then aggregated to derive a C stock difference between grasslands and native ecosystems representative of both MRT and MRH systems in the study region. The resulting values were 31.5 ± 6.3 and 30.9 ± 6.2 Mg C ha^−1^ for MRT and MRH systems, respectively. These values are in agreement with the estimates provided by Carter et al.^86^. LUC emissions from grassland and cropland expansion at LPS level were calculated based on the total amount of land undergoing the given transition in any 1 year, and the amount of CO_2_ emitted, after amortization, per unit of land for that LUC transition.

### Scenarios

This study explored three scenarios of improved feeding practices with and without feed crop yield improvements suitable to the agroecological conditions of southern and eastern Tanzania and for each dairy population (indigenous and improved). Similar scenarios were tested previously for Kenya by Brandt et al.^24,25^. This study modifies the scenarios to the policy context and priorities and to the best practice recommendations for the dairy sector in Tanzania (Table 2).

**Table 2:** Definitions of scenarios examined and their target populations of cattle.

Sector	Cattle population	Feeding strategy	Scenario abbreviation	Description
**Traditional**	Indigenous	Conservation	*L-Cn*	All maize stover fed to cows is treated with urea-molasses
		Conservation plus forage quality	*L-CnFo*	*L-Cn* with Napier grass increased to 25% of feed on offer, replacing grass and pasture
		Conservation plus forage quality with supplementation	*L-CnFoCo*	*L-CnFo* with 2 kg day^−1^ of concentrates fed during early lactation, and 0.5 kg day^−1^ during other periods. Concentrate intake is comprised of 67% maize bran and 33% sunflower cake
**Modern**	Improved	Conservation	*I-Cn*	All maize stover fed to cows is treated with urea-molasses
		Conservation plus forage quality	*I-CnFo*	*I-Cn* with Napier grass increased to 50% of feed on offer, replacing grass and pasture
		Conservation plus forage quality with supplementation	*I-CnFoCo*	*I-CnFo* with supplement feeding involving 5.0 kg day^−1^ of concentrates during early lactation, and 1.5 kg day^−1^ during other periods Concentrate intake is comprised of 67% maize bran and 33% sunflower cake

Under the strategy ‘Conservation’ (*Cn*), urea-molasses treated maize stover was fed to cows in place of untreated maize stover. A urea-molasses treatment is proposed to enhance the nutritional quality of stovers^12^. Therefore, in the dry season when availability and nutrient quality of forages is reduced, feeding treated maize stover can increase protein intake. The ‘Forage’ strategy (*Fo*) evaluated the role of higher rations of Napier feeding, in place of grass and pasture. For the ‘Concentrate’ strategy (*Co*), supplemental concentrates were provided to cattle according to supplementing regimes aimed at optimizing milk yields for local and improved cattle^31,87^. The choice of concentrate was based on Bwire and Wiktorrson^87^ who evaluated the effects of supplementing 67% maize bran and 33% sunflower cake rations on the performance of crossbred cattle in Tanzania. The concentrate and forage rations for improved cows were higher to meet their higher feed conversion efficiency^88^ (Table 2). All three of these strategies were evaluated additively by first implementing the conservation strategy, then assessing the additional effect of *Fo* and *Co*. This is because feeding greater concentrates was not found to be effective in improving milk yields unless seasonal feed deficits were first reduced (e.g. by using feed conservation and greater forage quality). For the results of additional scenarios, and the seasonal variation in nutrient availabilities for the cow simulations, see SI Sect. 6.

The Tanzanian Grazing-Land and Animal Feed Resources Act^89^ seeks to catalyse the development of Tanzania’s commercial feed processing industry. The simulations therefore focussed on yield gains in maize and sunflower for concentrate production, which are the two most common sources of concentrate feeds in the region^22^. Current yields of these crops (Table 1) are significantly below their potential, with water limited yield potential having been reported up to as high as 6.0 (maize) and 3.0 (sunflower) Mg ha^−1^ year^−1^^90,91^. Data from field experiments in Western Kenya^92^ were used to estimate the effect of higher N fertilizer application on yields and N_2_O emissions of maize and sunflower used in concentrate production. The yield gains were set as 50% of the yield gap based on the values reported above and in Table 1. The fertilizer requirement used to achieve these yields were based on an N-yield response of 14 kg ha^−1^ kg N^−1^, with an emission factor of 0.015 kg N_2_O kg N^−1^^92^. These scenarios were implemented in addition to the above feeding strategies, and denoted with a ‘+ *Cyg*’ (‘*Crop yield gains*’). The results of the yield gap and N_2_O calculations used for these simulations are shown in SI 4.

#### Baseline production growth and greenhouse emissions

A baseline provides a reference level against which a mitigation goal can be established^10^. The production practices used in the baseline represent those in the absence of specific mitigation interventions^93^. The dairy herd population for 2020 was established using spatially-explicit data on livestock population densities^94^ and annual growth rates in herd size. Feeding practices were obtained from GLS^33^ (SI 1). Model parameters for the *Baseline* were thus set by extrapolating historical values over the 10-year timeframe of the assessment. Throughout the 10-year simulation period, the herd size was assumed to grow by 5.5% and 4.5% annually for local and improved cattle, respectively^73^. No changes were assumed for feeding or other herd management practices that would otherwise affect productivity or herd compositions. The yields of feed crops were assumed to grow consistently with historical averages of 3.4% and 4.1% annually for maize and sunflower, respectively^40^. The scenarios were run modifying the availability of feeds, with and without yield improvements. For these scenarios, the populations and herd structures remained constant. The scenarios described above for both Traditional and Modern systems were thus run to compare to the *Baseline* scenario. This resulted in a total of 14 runs (2 baselines + 2 sectors × 3 feeding scenarios × 2 crop yield variants) for each LPS.

### Uncertainty assessment

Uncertainty in GHG emissions was quantified in line with the IPCC^29^ Guidelines. In the baseline, the sources of uncertainty were dairy cattle numbers per LPS, feed on offer per head, biomass yields, and emission factors (including coefficients on LUC transitions). For subsequent simulations the dairy herd and feed intakes were specified in relation to the baseline, and therefore for all other scenarios the only sources of uncertainty were in emission factors and biomass yields. Monte Carlo (MC) simulations were run for the baseline and each subsequent scenario to estimate the GHG emissions error range at a confidence interval of 95%. The standard error in emission factors were specified based on IPCC^29^ Guidelines. The uncertainty in the emission factor for enteric fermentation (Y_m_), which was calculated using Tier 3 guidelines, was set at 10%, consistent with previous studies estimating Y_m_ using Tier 3 guidelines^95^. The coefficients for LUC were calculated from country specific inventory studies and thus were either Tier 2 or 3 emission factors^48,49^. Moreover, because these coefficients were highly dependent on the C density data reported by Mauya et al.^49^, who report relatively low uncertainty (0.9% for forest and 1.8% for non-forest land), the standard errors for such were set at 20%. Because this study included simulations for greater N-fertilizer application, which may result in highly variable and uncertain changes in N_2_O emissions, the standard error of this emission factor (EF_1_ soil N inputs) was set at greater than double the required upper range for Tier 1 emission factors, taking a value of ± 66%. All other emission factors ranging from Tier 1 to 3 were set based on IPCC guidelines, thus ranging from 7 to 30% (SI 5).

## Results

### Evaluation of the baseline

**Figure 3:**
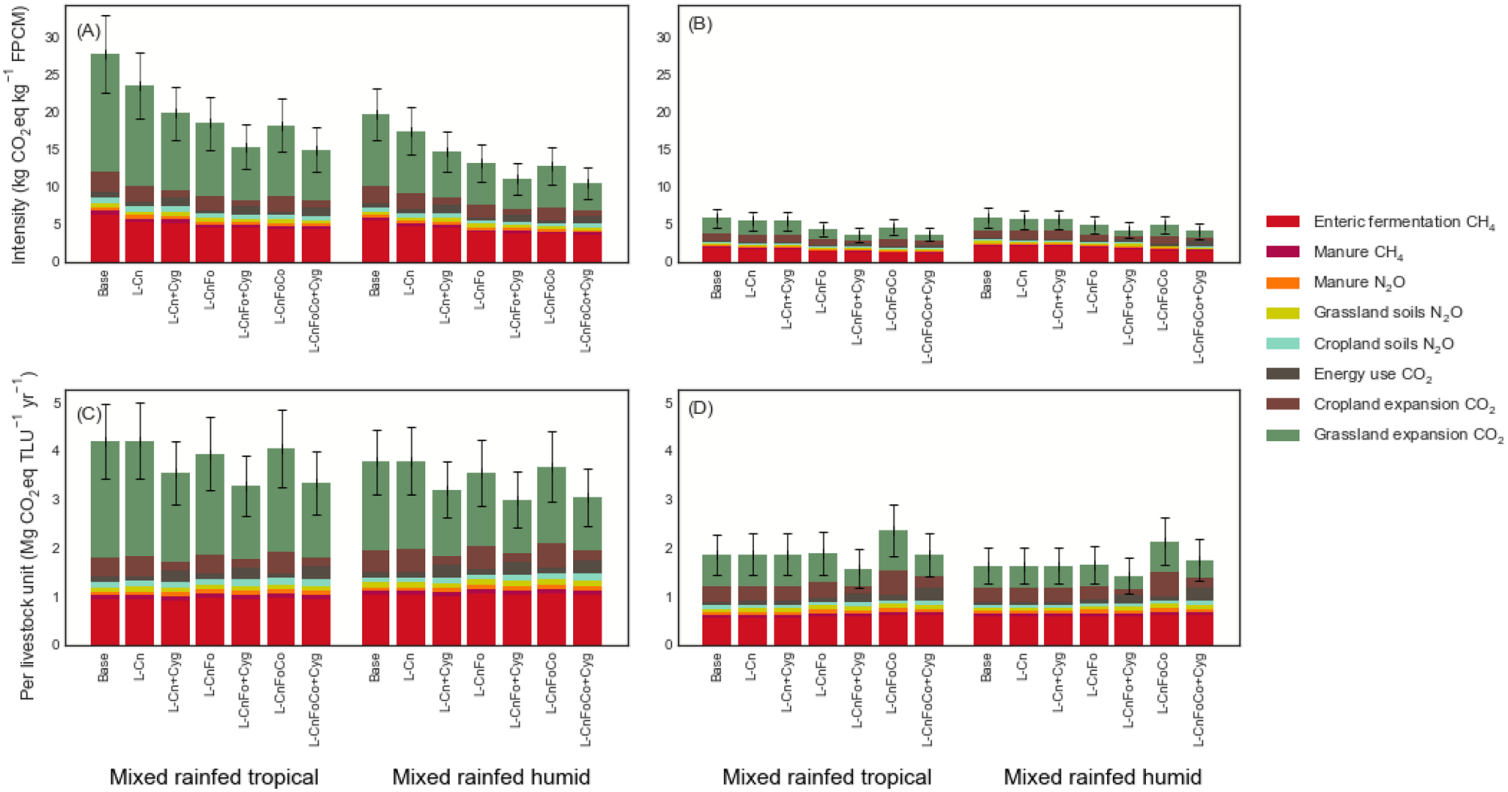
Greenhouse gas emissions for Traditional (**A**, **C**) and Modern (**B**, **D**) dairy sectors.

Direct emissions intensity (excluding LUC emissions) for the baseline were 9.3 ± 1.7 (95% confidence interval) and 7.8 ± 1.4 kg CO_2_eq kg^−1^ FPCM (MRT and MRH, respectively) for the Traditional sector. For the Modern sector, these emissions were 2.8 ± 0.62 and 3.2 ± 0.72 kg CO_2_eq kg^−1^ FPCM (MRT and MRH, respectively) (Fig. 3A,B). Emissions from LUC, expressed as emissions intensities, were 18.5 ± 4.1 and 12.0 ± 2.6 kg CO_2_eq kg^−1^ FPCM (MRT and MRH, respectively) for the Traditional sector and 3.0 ± 0.81 and 2.6 ± 0.57 kg CO_2_eq kg^−1^ FPCM for the Modern sector. The CO_2_ emissions from LUC (*cropland* and *grassland expansion*) throughout the simulation period (2020–2030) contributed between 45.8 and 65.8% of the total GHG emissions from milk production. Of the total LUC emissions, 7.7 and 29.2% (2.6 and 2.4 for MRT and MRH Traditional, and 0.98 and 0.81 kg CO_2_eq kg^−1^ FPCM for MRT and MRH modern sector, respectively) were from *cropland expansion*. The remaining 70.8–92.3% (18.5 and 12.0 for MRT and MRH Traditional, and 2.0 and 1.60 kg CO_2_eq kg^−1^ FPCM for MRT and MRH Modern sector, respectively) were from *grassland expansion*. The difference in LUC emissions between MRT and MRH is attributable to (a) a higher percentage of *grassland expansion* in MRT resulting in the conversion of native ecosystems, and (b) a larger land footprint for the dairy sector in MRT, owing to the larger herd overhead (i.e., the larger proportion of unproductive male and female cohorts in the herd, see herd composition by system in SI Table S1).

Since this study is the first quantitative assessment of GHG emissions that includes CO_2_ emissions from LUC from the Tanzanian dairy sector, these emissions estimates cannot be compared directly with other literature. However, using the Global Livestock Environmental Assessment Model (GLEAM), FAO New Zealand^14^ estimated direct emissions in Tanzania’s dairy sector, which included emissions from enteric fermentation, manure, N_2_O emissions from managed soils, as well as CO_2_ from feed and fertilizer production/transport. FAO New Zealand estimated emissions intensities from these sources within the range of 20–28 and 2–3 kg CO_2_eq kg^−1^ FPCM for the Traditional and Modern sectors respectively (including from both MRT and MRH systems). This latter study, which is a nationally representative study of Tanzania, estimated lower milk yields for local cattle (200 L per lactation). In the present study focussing specifically on mid to high productivity (i.e. excluding pastoral) systems in the southern highlands and Morogoro, yields were estimated at significantly higher levels (582 and 538 L lactation^−1^ for the MRT and MRH baselines, respectively). Hence, the direct emissions intensities were estimated to be 53.5–61.0% lower than those estimated by FAO New Zealand. The emissions intensities for the Modern sector of the present study are comparable to those of FAO New Zealand and those of neighbouring countries with a high proportion of crossbred dairy cattle (e.g., Kenya). In Kenya, emissions intensities have been estimated to be 2.2–3.0 kg CO_2_eq kg^−1^ FPCM^24,96^.

### Impact of feeding intensification on direct non-CO_2_ GHG emissions

Direct emissions intensities were reduced by up to 28.2 ± 5.1 and 29.2 ± 5.3% for local cattle in MRT and MRH, respectively (Fig. 3A). For improved cattle, the scenarios led to declines in direct emissions intensities of up to 28.0 ± 6.2 and 26.7 ± 5.9% (MRT and MRH) (Fig. 3B). The scenarios resulting in the largest declines in emissions intensities were the forage quality plus concentrates scenarios (*L-CnFoCo* and *I-CnFoCo*), and for the simulations without yield gains in feed crops. Since the diets for scenarios with and without yield gains were identical, the slightly higher value for direct emissions intensities for the yield gains scenarios was caused by an increase in soil N_2_O emissions from croplands by 16–40%, and in energy use CO_2_ by between 220 and 242%.

All the scenarios assessed for all systems led to greater intake of metabolizable energy and protein, which led to 18–52% and 6–63% gains in milk yields for cows in the Traditional and Modern sectors, respectively (Table 3). All the scenarios resulted in greater annual gross energy intake per cow, and while these represent modest declines in Y_m_, up to a maximum of 7.5%, the impact on CH_4_ emissions from enteric fermentation were negligible. Changes in enteric CH_4_ ranged between − 3.8 and + 8.7%. Manure CH_4_, also because of higher gross energy intake, increased by up to 15.4%. Manure N_2_O increased by up to 40.5%, because of the higher protein concentration of the diets and consequently higher N excretion in manure. The only scenarios that did not lead to higher manure CH_4_ was *Conservation* (*Cn*). In summary, the scenarios therefore resulted in modest increases in absolute GHG emissions from enteric fermentation, manure and soils, by between 0.0 and 14.1% (Traditional) and 0.0–33.1% (Modern) (Fig. 3C,D). However, through their impacts on milk yields, these scenarios had significant impacts in reducing emissions intensities, up to 29.2% (Traditional) and 28.0% (Modern). The scenarios thus improved emissions efficiency (emissions per unit FPCM), but they did not actually reduce direct non-CO_2_ emissions in absolute terms (i.e. per TLU).

**Table 3:** Effects of feeding scenarios on milk yield for the Traditional sector (local cattle) and Modern sector (improved cattle).

Scenarios	Feeding practices	Mixed rainfed tropical			Mixed rainfed humid		
		Milk yield			Milk yield		
		Lactation	Annual	Change	Lactation	Annual	Change
		(kg FPCM cow^−1^ lactation^−1^)	(kg FPCM cow^−1^ year^−1^)	(%)	(kg FPCM cow^−1^ lactation^−1^)	(kg FPCM cow^−1^ year^−1^)	(%)
**Traditional sector (local cattle)**							
Base	Baseline	582	358		538	331	
L-Cn	Feed conservation	689	424	+ 18.4	611	377	+ 13.9
L-CnFo	Feed conservation, forage quality	823	507	+ 41.6	758	466	+ 23.6
L-CnFoCo	Feed conservation, forage quality, concentrates	858	528	+ 47.4	813	501	+ 51.4
**Modern sector (improved cattle)**							
Base	Baseline	1413	932		1326	875	
I-Cn	Feed conservation	1458	991	+ 6.3	1387	915	+ 8.3
I-CnFo	Feed conservation, forage quality	1833	1264	+ 35.6	1580	1059	+ 25.3
I-CnFoCo	Feed conservation, forage quality, concentrates	2163	1492	+ 60.1	1965	1355	+ 54.9

### Land use effects of changes in feed mixes (not including crop yield gains)

**Figure 4:**
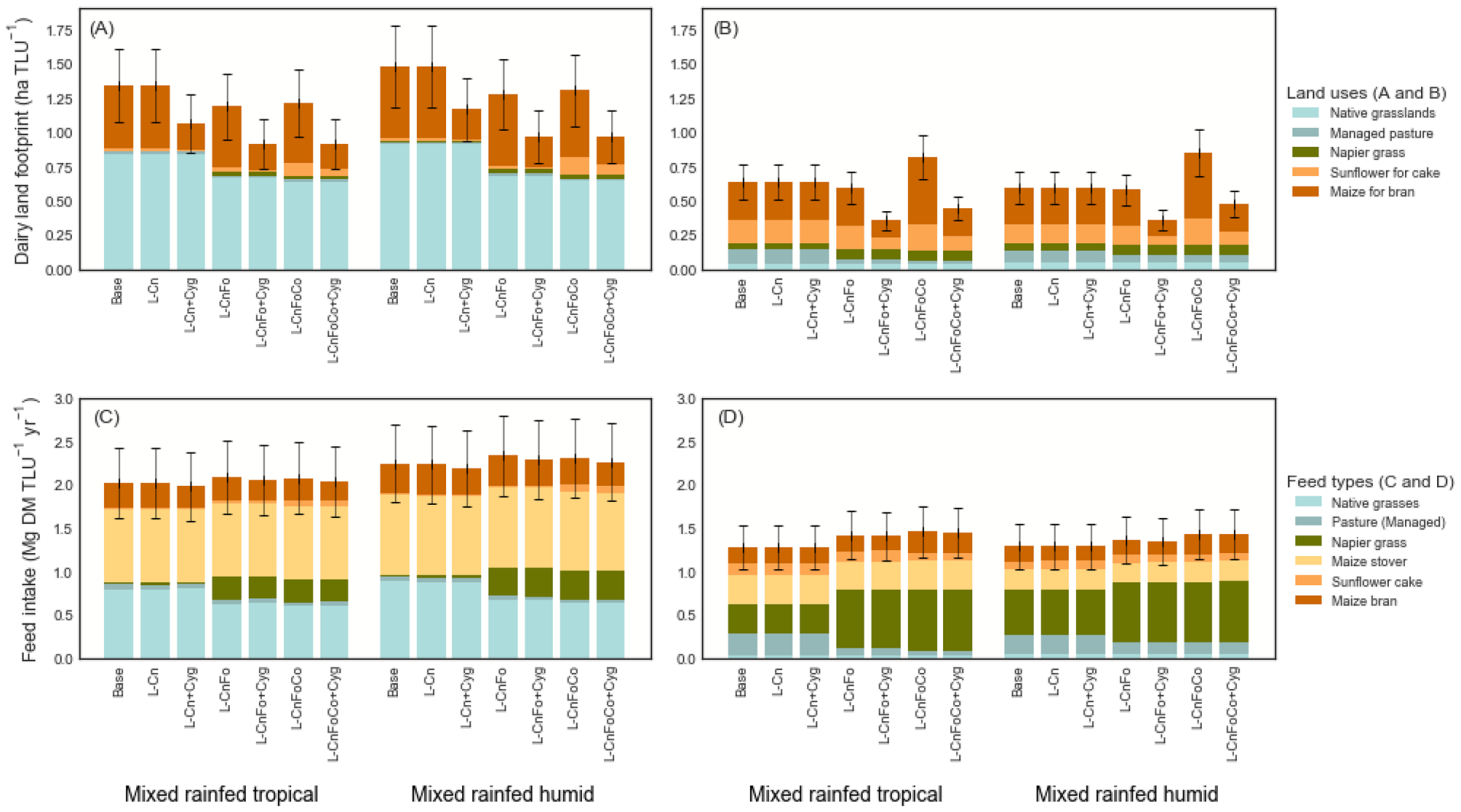
Dairy land footprint and feed intakes for Traditional (**A**,**C**) and Modern (**B**,**D**) dairy sectors.

The scenarios resulted in 4.6–45.0% greater cropland area and 17.6–28.9% less grassland area under use as part of the dairy land footprint (Fig. 4A,B). The scenarios *L-Cn* and *I-Cn* were exceptions as they did not result in LUC because this strategy only involved the treatment of available maize stover fed to cows. For the Traditional sector, dedicating greater area to feed crops under *L-CnFoCo* resulted in between 410.0 and 557.0% greater land under sunflower and 3.0–7.0% less land under maize (for concentrate production). For the Modern sector (*I-CnFoCo*), between 15.0 and 37.0% greater maize and 75.2–82.2% greater sunflower areas resulted from the increase in concentrate feeding. These scenarios consequently resulted in between 2.0 and 11.5% (Traditional) and 52.0–66.5% (Modern) greater CO_2_ emissions from *cropland expansion* relative to baseline. Concurrently, the land areas required for grasslands declined by between 21.0 and 25.7% (Traditional) and 29.0–29.4% (Modern).

The net effect of these changes was a reduction in the dairy land footprint by 7.4–9.5% and 6.1–8.2% for the *L-CnFo* and *L-CnFoCo* scenarios, respectively, for the Traditional sector. For the Modern sector, *I-CnFo* and *I-CnFoCo* led to 30.1–32.5% less and 20.9–31.8% greater land footprints, respectively. The increase in cropland area dedicated to concentrate feeds crops under *I-CnFoCo* outweighed the decline in grassland area and hence the total land footprint increased (Fig. 4B,D, I*-CnFoCo*). These changes resulted in reductions of between 8.0 and 31.1% (Traditional) and 10.9–16.0% (Modern) in emissions associated with *grassland expansion*. Under *I-CnFoCo*, while the land footprint increased, only between 29.8 and 49.5% of this additional area expansion resulted in the conversion of native ecosystems. Therefore, for all scenarios there were reductions in total LUC CO_2_ emissions, by 7.2–15.5% for the Traditional sector and 1.2–4.1% for the Modern sector.

#### Effects of crop yield gains on the land footprint and GHG emissions

The fertilizer-induced yield gains in maize (for bran) and sunflower (for cake) led to an increase in soil N_2_O emissions by a factor of 5.5 for maize and 3.2 for sunflower (full results in SI Sect. 4). These increases occurred concurrent with a 2.25 and 1.0 Mg ha^−1^ year^−1^ increase in the yields of these crops. Hence, absolute N_2_O emissions per hectare for these two crops, as well as yield-scaled N_2_O emissions, increased. These yield gains however led to less area of these two crops needed to satisfy the feed demands for the dairy herd. Relative to the scenarios without yield gains, the total area dedicated to maize (for bran) and sunflower (for cake) declined by 57.6 and 47.4%, respectively (Fig. 4A,B), as a result of these yield gains. Moreover, most of the scenarios (with the exception of the feed conservation scenarios) involved the substitution of feeds with relatively low soil N_2_O emissions (native grasslands) for feeds which have relatively high N_2_O fluxes (Napier grass and concentrate feed crops) (Table 1) (Fig. 4C,D). Therefore, the fertilizer-dependent yield gains have the effect of increasing total N_2_O emissions relative to the scenarios with the same diets with baseline yields for concentrate feeds. Moreover, while the dietary impact of these changes was higher milk productivity (Table 3), the growth in milk production is not sufficient to lead to an actual decline in the soil N_2_O emissions intensity. Relative to the baseline crop yield growth variant, N_2_O emissions intensities therefore rose by a maximum of 34.0%. The additional reliance on concentrate feeds also led to greater CO_2_ emissions from energy use upstream from the farm, increasing by between 220 and 232% (Traditional sector) and 227–246% (Modern sector). This also led to higher CO_2_ emissions from energy use per unit of milk. However, despite the growth in N_2_O and CO_2_ emissions from crop yield gains, these have the effect of reducing LUC emissions, both from *cropland expansion* (e.g., because less crop area was required to meet the crop feed demands) and from *grassland expansion*. The latter occurred because the yield gains in feed crops implied less grasslands needed to be converted to cropland to satisfy the crop feed demands, and hence there would be less expansion of grasslands needed to replace the grassland converted to croplands. In summary, the fertilizer-dependent yield gains have the effect of increasing N_2_O emissions from soils and energy use CO_2_, both in absolute terms and per kg FPCM. However, the decline in land converted to cropland due to improved yields would result in less *cropland* and *grassland expansion*, and thereby lower LUC emissions. The reduction in LUC emissions outweighed the increase in emissions from soils and energy use, and therefore in net terms, the crop yield gains reduced GHG emissions attributable to milk production by between 11.4 and 4.4% (Traditional) and 29.5–34.9% (Modern).

## Discussion

To the knowledge of the authors, this study presents the first comprehensive assessment of GHG emissions from Tanzania’s dairy sector that includes the impact of indirect emissions from expanding crop and grassland areas. Initiatives to include the dairy sector in Tanzania’s NDC or, for example, to develop a dairy NAMA will require foresight analyses, which provide empirical evidence quantifying the impact of proposed mitigation strategies on GHG emissions and on milk productivity. This study therefore offers the first assessment of such dimensions, which can be used in subsequent analyses that consider additional mitigation strategies (e.g. animal genetic gains)—also in conjunction with cost–benefit analyses. It thereby supports ongoing public and private efforts to formulate evidence-based mitigation strategies available.

The framework used in this study, based on principles of attributional life cycle assessment, was instrumental in showing how LUC emissions are comparatively significant in relation to direct non-CO_2_ emissions. These account for 45.8—65.8% of total GHG emissions from the dairy sector. Because all the scenarios resulted in increases in direct non-CO_2_ emissions by between 0.6 and 33.1%, our analysis demonstrates that emissions from LUC deserve to be prioritized in future mitigation strategies. Importantly, this study highlights that reducing the dairy land footprint through improved feeding practices combined with crop yield gains has particular mitigation potential by curbing emissions from cropland and/or grassland expansion. These results could be used to guide the development of a dairy NAMA or the refinement of the NDC benefiting from synergies resulting from improved feeding practices and crop yield gains on dairy sector productivity and land use. The higher milk yields would result in economic benefits for dairy producing households across the four studied regions. The milk productivity and GHG emissions estimates described above could help stakeholders who must balance both environmental and socioeconomic criteria in designing climate change mitigation policies (Lin et al.)^97^, and who must target populations based on criteria such as breed of cattle owned (i.e. the Traditional or Modern sectors, as described above) or on productivity potential across livestock production systems.

This study contributes to the knowledge base by providing GHG mitigation potential at sub-national scale from reduced land use in the dairy sector, a first for sub-Saharan Africa (SSA). Previously, Brandt et al.^25^ evaluated comparable feed and crop yield scenarios in Kenya using a framework that included CO_2_ emissions from cropland expansion as well as forest grazing. The present analysis includes both *cropland* and *grassland expansion* using longitudinal simulations and the feeding and crop yield scenarios were evaluated in relation to a baseline. This study found that avoided emissions from *grassland expansion* were the main cause of emissions reductions. These emission reductions were more significant (declining by up to 1.2 and 12.9 kg CO_2_eq kg^−1^ FPCM for Modern and Traditional sectors, respectively) than the estimated reduction in CO_2_ emissions from forest grazing by Brandt et al.^25^, estimated at 0.06 kg CO_2_eq kg^−1^ milk under the optimal feeding and maize yield scenarios. Similar as the present study, other top down, regional studies using the Global Biosphere Model (GLOBIOM) (Havlik et al.)^84^ have found that land sparing could be a key mitigation strategy in the beef and dairy sectors^27,98^. Gerssen-Gondelaach et al.^27^ calculated that LUC-related emissions across Latin America, South-East Asia, and Sub-Saharan Africa (SSA) account for 20 to over 50% of total GHG emissions from beef and dairy production systems, and suggest that reducing LUC-related emissions is a key strategy for reducing GHG emissions from dairy production in SSA and other regions.

The realization of avoided LUC emissions could be influenced by a demand or supply rebound^99^. Based on the partial equilibrium analysis of Valin et al.^100^, improving productivity in ruminant productions systems in SSA under the presence of highly elastic demand for milk and dairy products resulted in production rebounds sufficient enough to negate emissions savings from reduced LUC. In Tanzania, increasing domestic milk production is a pillar of the national poverty alleviation strategy^7^ and therefore policy initiatives in the dairy sector will likely favour continued supply growth, by improving availability of inputs, promoting improved production practices, and further developing dairy supply chains^7^. Such factors combined with increasing demand from a growing and increasingly affluent and urbanized population, or from increased demand from trading partners, could result in significant growth in production in coming years. This thus poses the risk that efficiency gains result in greater crop and grazing land expansion, increasing CO_2_ emissions from LUC, leading to similar outcomes as well documented cases in South America^100^. We caution therefore that more work is needed to evaluate the risk for these outcomes. For evaluating these outcomes, consequential LCA is more suitable than the attributional method used here, owing to its ability to account for indirect land use change, import substitution and substitution between beef and milk production^101^.

### Prioritizing climate change mitigation activities in Tanzanian dairy

Since LUC emissions comprise a large portion of the C footprint, it logically follows that changes that lead to a reduced land footprint, such as by replacing low yielding native grasslands (≤ 3 Mg ha^−1^ year^−1^) with Napier grass (≥ 10 Mg ha^−1^ year^−1^) or through yield gains in feed crops, could result in avoided emissions from LUC. However, this study did not find strong evidence that feed intensification in itself contributes to avoided LUC. It attributes this to the effect of increases in crop-based feeds (maize bran or sunflower cake) on land use (scenario *I-CnFoCo*), which led to a larger land footprint. The dietary changes under this scenario brought the level of concentrate intake to levels reminiscent of intensified dairy farms. For example^96^, reports that dairy farms in Kenya typically use 1–2 kg cow^−1^ day^−1^ of concentrates. Thus, based on these results, we caution that adoption of improved feeding practices, insofar as these lead to greater demand for feed crops, have potential to increase the dairy land footprint, leading to higher CO_2_ emissions from LUC. However, the present analysis also shows that crop yield gains can offset this. This has a net negative effect on the overall carbon footprint because N_2_O and CO_2_ emissions from crop yield gains is low relative to the avoided emissions from LUC. Although the present study only assumed a 50% yield gap reduction, it still estimated emissions savings that are 105% larger than those estimated by^25^ (this study simulated crop yield gains of up to 80% of the water limited yield potential for maize). The higher estimated net mitigation of the yield gains herein were attributable to the inverse relationship with area of grassland under use for feeding, which in turn translated into reduced conversion of native ecosystems. We therefore expect that initiatives under the Tanzanian Grazing-land and Animal Feed Resources Act^89^, as well as complementary programs in the grain and oilseed subsector^102^, could result in mitigation co-benefits for the dairy sector. In order to maximize the likelihood of co-benefits, these policy initiatives should promote best practices to increase the yields of feed crops. Such practices would sustainably enhance yields and minimize N_2_O fluxes resulting from application of N-fertilizer^103^.

The feeding strategies evaluated for the Traditional sector suggest that reducing seasonal feed deficits are essential in improving emissions efficiency of this sector. Feeding high quality forages or concentrates will not result in improved productivity unless seasonal feed deficits are better managed since poor body condition caused by periodic feed deficits can have lasting effects on milk productivity and reproduction^105^ and lifetime productivity of the cows^31^. Of the scenarios evaluated above and the additional scenarios presented in SI 6, feeding additional concentrates during lactation was not found to be particularly effective if a feed conservation strategy was not first implemented. Based on this we propose that dairy farmers rearing local cattle (the Traditional sector) should be supported to adopt better feed conservation practices, such as treatment of stovers, or of silage or hay making practices. Such practices would improve productivity by reducing dry season milk yield shortfalls. While treatment of stover is relatively safe and easy, lack of access to urea is often cited as a constraint to widespread adoption of this practice^12^.

The benefits of higher milk yield and lower emissions intensities from improved feeding will be highest when these interventions are targeted to the Modern sector. Moreover, owing to the higher feed conversion efficiency and greater efficiency of *Bos taurus* genetics^88^, a greater uptake of *Bos taurus* in place of *Bos indicus* genetics could allow for milk production targets to be met with a smaller land and carbon footprint. Notenbaert et al.^26^ evaluated the role of genetic gains on GHG emissions and household food security in the Tanga region of Tanzania, estimating that genetic gains could reduce emissions intensity of milk by as much as 50%. However, their study only accounted for direct non-CO_2_ emissions, and thus potentially omitted a significant component of the dairy C footprint occurring from LUC. Based on our estimates, the Traditional sector, due to the greater reliance on native grasslands and the comparatively large herd overhead (larger proportion of unproductive cattle), has more than twice the land footprint (1.25–1.50 ha TLU^−1^ versus 0.60–0.70 ha TLU^−1^) and up to a 4.5 higher C footprint, when the role of LUC emissions are accounted. We emphasize therefore that genetic gains offer significantly larger GHG mitigation potential than previously estimated. It is proposed that because the Traditional sector is constrained by low feed conversion efficiency and contributes the majority of the LUC emissions, genetic gains should be a priority focus for GHG mitigation initiatives. Genetic gains would help to capitalize on synergies resulting from improved feeding and animal husbandry, and will be more effective at reducing emissions intensities when combined with yield gap reductions in the feed crop sector. In this regard, there can be synergies between these GHG mitigation initiatives and existing priorities under the LMP, for which genetic gains and feeding practice improvement are key components^8^.

### Feeding management in Tanzania’s livestock master plan and GHG emission targets

The milk yield gains in our scenarios are as high as 51.4% and 60.1% for local and improved cows, respectively. These milk productivity gains were associated with up to 52.4% and 38.0% declines in emission intensities in the Traditional and Modern sectors, respectively. Using the baseline estimates of milk production from the above simulations, the estimated supply gap projected by the LMP^8^ of a factor of 71.0% of the national milk demand by 2030 could be reduced by up to 32.1%. Alternatively, if the milk supply gap were to be wholly eliminated, these changes in feeding practices would allow for a 33.3% reduction in the size of the dairy herd relative to a scenario involving baseline feeding practices. Such changes in feeding practice combined with the yield gap reductions simulated in this study would allow milk production targets to be met with up to 52.4 and 38.0% reductions in emissions intensities for the Traditional and Modern sectors, respectively.

### Limitations and suggestions for future research

#### Data limitations and modelling uncertainty

Emission factors (EFs) in this study are based on the best available estimates from the literature and values ranged from Tier 1 to Tier 3^29^. An advantage of the approach taken here was that the EFs that have the largest impact on the dairy sector’s GHG footprint (i.e. enteric fermentation and LUC) were calculated with Tier 2 and 3 factors. Central to the development of more accurate GHG accounting frameworks for crop and livestock production will be the availability of country specific EFs, such as those pertaining to emissions from manure management, and crop and grasslands. The same applies to datasets on livestock population densities, as well as data on feed ratios/intakes of livestock. The present study benefitted from the most recent gridded livestock of the world dataset^94^, which to the knowledge of the authors is the most accurate source of spatial data on livestock population densities currently available. The diets specified herein were based on survey data^35^, which is prone to erroneous farmer recall. Moreover, it is known that livestock diets vary highly across geographies and farm types. This introduces uncertainties in diet baselining. All these sources of uncertainty were nevertheless quantified in the present study through Monte Carlo simulations.

The LUC transition framework in this study was based on the assumption that cropland expansion converts grasslands, which may not always be the case. While this study did not consider management changes within a given land use category, the scenarios assessed were designed to reduce the requirement for grazing (e.g. by reducing the total grassland requirements), and therefore in principle should result in less demand for grazed biomass, and hence degradation of grasslands or native ecosystems. In this respect, the use of a dynamic livestock model was instrumental, because the change in roughage intake with changing dietary regimens is explicitly accounted for. The further development of methodologies for accounting for the impact of grazing practices on land degradation and LUC, and for validating these methodologies on the ground, will assist studies such as ours with the development of region- or country-specific GHG emission estimates.

#### Suggestions for future work

The modelling framework developed for this study is publicly available (see data availability below) and thus other researchers working at the intersection of dairy production and climate change mitigation could extend this analysis further. Extending the framework in this study using a consequential LCA would be warranted given the greater depth and policy insights provided by this over the attributional method. Examining other mitigation strategies is also warranted, especially genetic gains, animal husbandry (health and reproductive practices) and land management (e.g. grazing practices) which have been not been included here. Future studies should aim to refine and achieve greater consensus as to the role of LUC in dairy GHG mitigation in low income and tropical regions and how to account for these changes in LCA, for which there remains relatively little existing literature and wide deviations in findings. Greater consensus in this regard will help inform the effective design of climate initiatives at national levels, for which, in the SSA region, LUC is known to play a critical role^27,100^. Future work to evaluate LUC emissions reductions specifically from the above listed mitigation strategies would advance knowledge as to synergies between these different practices and technologies and help inform climate policy in the region.

## Conclusion

This study assessed the GHG emission and national milk deficit reduction potential of improved feeding practices and feed crop yield gains in Tanzania’s south/eastern regions. Changes in feeding practices involving feed conservation, the addition of high quality forages to diets, and concentrate feeding, combined with crop yield improvements, have potential to reduce the dairy sector’s land footprint concurrent with reductions in GHG emissions intensities by up to 52.4% in the Traditional and 38.0% in the Modern sectors. These changes in practices can increase milk productivity by up to 60.1% and 51.4% for local and improved cows, respectively. While the feeding strategies evaluated in this study may potentially result in greater LUC emissions, a key finding was that fertilizer-induced yield gains in primary concentrate feed crops lead to net reductions in the C footprint of the dairy sector. These results therefore demonstrate the impacts of the potential feeding options and/or crop sector initiatives, which can be used alongside dairy genetic gains in order to meet the milk production and national GHG mitigation targets.

## Supplementary Information

Supplementary Information.

## Data Availability

The data that were used to parameterize the model and run the simulations are described and presented in the text and “Supplementary information” to this paper. The algorithm used to run the livestock model, conduct the life cycle assessment, and spatial aggregation is available as python code from https://github.com/JamesHawkins/sectoral_land_model_Tanzania_data.
