# Author Correction: Feeding efficiency gains can increase the greenhouse gas mitigation potential of the Tanzanian dairy sector

**DOI:** 10.1038/s41598-021-89524-6

**Published:** 2021-05-03

**Authors:** James Hawkins, Gabriel Yesuf, Mink Zijlstra, George C. Schoneveld, Mariana C. Rufino

**Affiliations:** 1 Lancaster Environment Centre, Lancaster University, Lancaster, UK; 2 Plant Production Systems, Wageningen University, Wageningen, The Netherlands; 3 Center for International Forestry Research (CIFOR), Bogor, Indonesia

Correction to: *Scientific Reports* https://doi.org/10.1038/s41598-021-83475-8, published online 18 February 2021

The original version of this Article omitted the Acknowledgements section. The Acknowledgements section now reads:

“This research was in part funded by the IFAD project “Greening livestock: Incentive-based interventions for reducing the climate impact of livestock production in East Africa”, as a contribution to the CGIAR program on Climate Change, Agriculture and Food Security (CCAFS).”

The original Article has been corrected.

