# Supplementary material for: Feeding efficiency gains can increase the greenhouse gas mitigation potential of the Tanzanian dairy sector
Source: Sci Rep. 2021 Feb 18;11:4190. doi: 10.1038/s41598-021-83475-8 (PMC7893068; doi:10.1038/s41598-021-83475-8)
Supplement: Supplementary file 1 — Supplementary Information. [file 41598_2021_83475_MOESM1_ESM.docx]

**Supplementary Information**

Feeding efficiency gains can increase the greenhouse gas mitigation potential of the Tanzanian dairy sector

Authors:

James Hawkins^1^

Gabriel Yesuf^1^

Mink Zijlstra^2^

George C. Schoneveld^3^

Mariana C. Rufino^1^

**1. Livestock simulations**

LivSim is a dynamic model that simulates the performance of individual cattle in time according to their genetic potential and feeding ^[32]^. Its development and applications have focussed on assessing the impact of productivity improvement among dairy production systems in the tropics, which are characterized as having highly variable quality and availability of feed across seasons. The model has been validated and applied in studies ranging from farm scale to sector level, and spanning both East and West Africa ^[32] [25] [81]^ . Inputs to the model include breed characteristics, feeding, and other animal husbandry practices which influence productivity and nutrient requirements (grazing practices, reproduction management). In the present framework, the outputs of the model pertaining to feed intake from feed on offer, average annual milk yield over the production life of the cow, and urinary and faecal N excretion were used as the basis of the LCA and productivity evaluation (Figure S1) . Based on the feed intake and N excretion, CH_4_ and N_2_O emissions from enteric fermentation and manure were estimated, thus providing the direct emissions from milk production used in the LCA (SM Section 2). The diet compositions as estimated from GLS (2019), taking into account the biomass yields of individual feed categories (Table 1 of text), were used to derive the land footprint for the dairy sector, using equation 1 in methods. This land footprint was the basis for specifying feed on offer every month of the year, based on the feeding practices as specified below. The amount of land dedicated to crop and grasslands was then used for calculating land use change emissions as described in section 2.3.


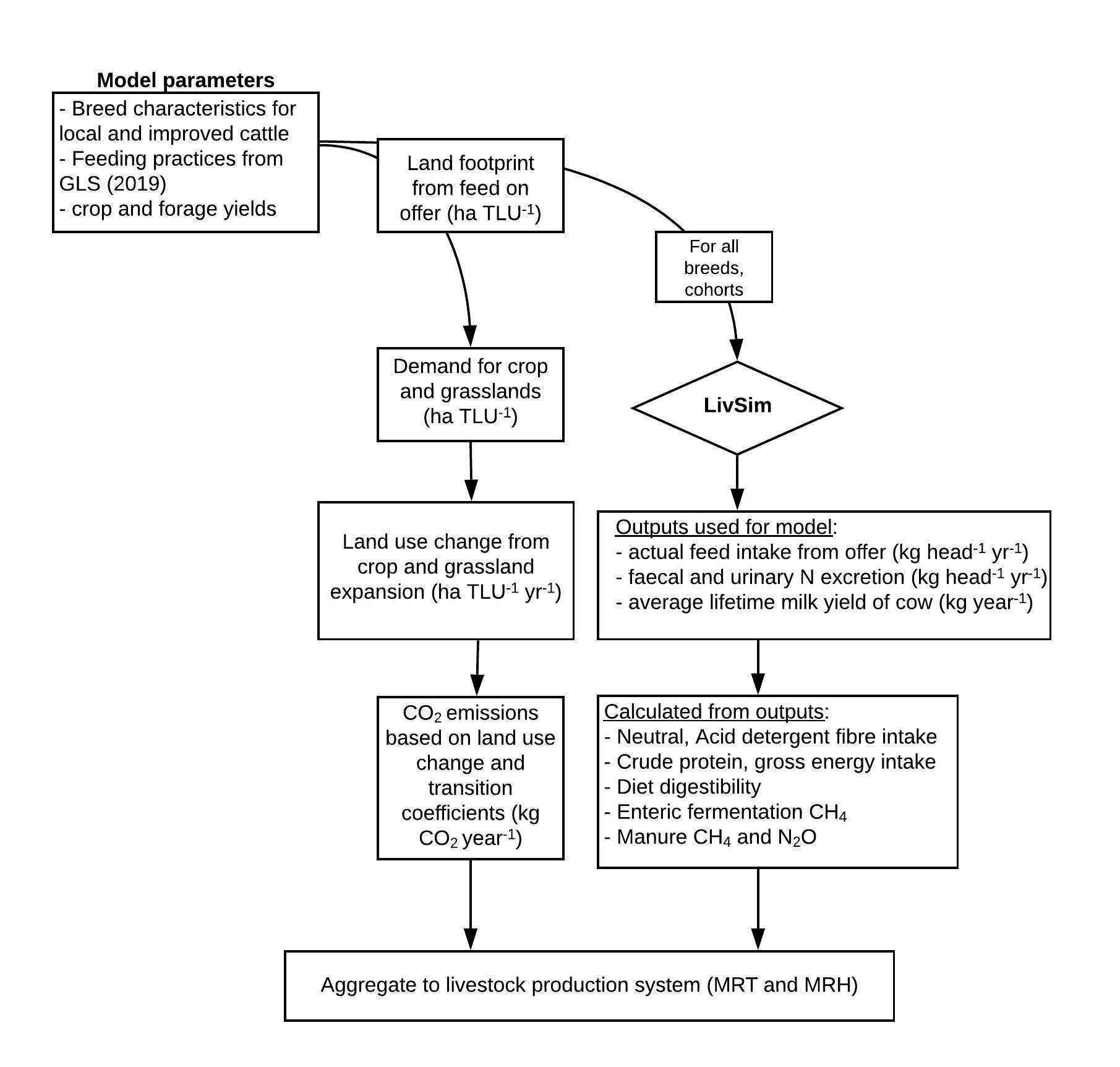


Figure S1: Schematic flowchart of the modelling framework, integrating LivSim with an accounting of the dairy land footprint, life cycle assessment of GHG emissions, and spatial aggregation to production system level (MRT and MRH)

Parameters obtained from a variety of sources in the literature were used to specify breed parameters representing local and improved cattle in Tanzania (Table S1). The activity allowances were set reflecting the amount of grazing time. All animals (both local and improved) are typically kept in corrals at night and grazed during the day. GLS (2019) indicates that improved cattle are typically grazed for less than 2 hours per day. Local cattle are typically grazed for 6 hours or more per day. The feed intake, milk production and excretion results were determined as an annual average calculated over a pre-defined age range for each cohort and breed. These ranges were (for each respective cohort): male and female calves, 0 months to 1 year; juvenile males, 1 to 3 years; heifers, 1 year until first calving; cows, from the beginning of the first calving onwards; and bulls, 3+ years.

The results of the breed and cohort simulations were aggregated to production systems based on the respective cattle populations for each system (MRT and MRH). The populations of cattle by breed and cohort were specified based on a spatially-explicit dataset of cattle population densities (e.g. head of cattle per sq. km.) ^[70]^.The ratio of ‘dairy cattle’, which includes the local and improved breeds described in the text, to the total population (per sq. km) reported by ^[70]^ were equal to the total value minus the fraction of beef cattle and oxen, as determined from district census data ^[71]^.The fraction of total dairy cattle categorized as local or improved was also based on district level census data ^[71]^.The herd compositions for a given breed (i.e. the proportion of total animals in a given cohort: cows, heifers, calves, etc.) were derived from the survey (GLS 2019), as an average value for each LPS (Table S2, percentage of cattle for each LPS). This data was then mapped onto spatially explicit datasets at 10x10 km resolution of MRT and MRH production systems and then up-scaled to estimate total cattle populations by breed and cohort at the production system level (Table S3). The spatial analysis and upscaling was performed in Qgis ^[82]^.

Table S1: Breed parameters used in LivSim

| Parameter | Local | Improved | Source |
| --- | --- | --- | --- |
| Maximum body weight female (kg head^-1^) | 450 | 600 | [46]  [91] |
| Maximum body weight male (kg head^-1^) | 500 | 600 | [46]  [91] |
| Maximum milk yield (kg lactation^-1^ cow^-1^) | 970 | 4450 | [48]  [92] |
| Daily milk yield at maximum (litres) | 8 | 15 | [92]  [93] |
| Lactation length (days) | 210 | 300 | [46]  [95] |
| Milk fat content (g kg^-1^) | 55 | 41 | [96] |
| Milk crude protein content (g kg^-1^) | 41 | 35 | [96] |
| Calf birth weight (kg) | 30 | 32 | [97] |
| Minimum age at first gestation (months) | 30 | 20 | [95]  [98] |
| Pregnancy length (months) | 9 | 9 | [46]  [95] |
| Dry period (months) | 11 | 2 | [46]  [99] |
| Postpartum length (months) | 12 | 3 | [46]  [99] |
| Maximum lifetime (years) | 13 | 13 | [32] |

Table S2: Herd populations by production system

| Breed/cohort | MRT | MRH |
| --- | --- | --- |
| Local (heads) | 603,808 | 458,307 |
| Cows (%) | 38.78 | 55.46 |
| Heifers (%) | 13.61 | 21.48 |
| Female calves (%) | 21.77 | 6.14 |
| Bulls (%) | 11.03 | 9.81 |
| Juvenile males (%) | 2.96 | 4.59 |
| Male calves (%) | 11.86 | 2.52 |
| Improved (heads) | 19,926 | 15,124 |
| Cows (%) | 49.41 | 45.38 |
| Heifers (%) | 11.79 | 15.99 |
| Female calves (%) | 20.01 | 18.99 |
| Bulls (%) | 6.34 | 7.80 |
| Juvenile males (%) | 2.01 | 3.38 |
| Male calves (%) | 10.23 | 8.46 |

*Specifying feed on offer for LivSim*

The method of specifying feed on offer per month for each livestock category involved two steps. First, the household survey was used with supplementary datasets of feeding in the southern highlands region of Tanzania to estimate the annualized feed intake of the broad feed categories (Table 1 of text) per year for each animal in the herd. This annualized value takes into account the deviation in feed intakes across dry and rainy seasons. Then the availability of these feeds for every animal across months (feed on offer for LivSim) were specified taking into account the major factors influencing seasonality of each feed category, as described below.

The survey questionnaire disaggregates feed categories into concentrates, by-products, crop residues, improved forages and low quality forages. The intake levels that were derived for each category were used as the basis for the baseline feeding practices in the model. ‘Sunflower cake’ was the feed representing the level of concentrates fed. ‘Maize bran’ was used as the feed representative of crop by-products. Maize stover represented crop residues, Napier represented improved forages, and ‘Pasture’ represented the variety of cultivated low quality forages. For grass consumed from grazing, the species were specified as a mixture of the dominant grass species in Tanzania, *Themeda spp* and *Hyparrhenia spp* ^[23]^.

*Deriving feed intake from the dairy household survey*

GLS (2019) evaluates, based on the recollection of the survey respondent, the feed on offer from individual categories of feeds, obtained from on-farm and off-farm (market purchases) sources. In semi-intensive and extensive systems where cattle consume biomass while grazing, the biomass consumed from grazing was estimated and included as ‘grazed feed intake’, in addition to feed on offer from farm harvest and market purchases. This intake level was assumed to be at least as great as 2.5% of bodyweight. To estimate feed intake during the alternate season, parameters were derived from ^[92]^ to account for the differences in intake of feed categories between dry and rainy seasons. From these values, the total annual feed intake for the herd was then estimated based on the average intake over the dry and rainy seasons as follows:

Annual feed intake _i_ = 365 x $\frac{{Daily dry season feed intake}_{i} + {Daily rainy season feed intake}_{i}}{2}$ (1)

Where Annual feed intake is the annual feed intake for a given feed category f (kg TLU^-1^ yr^-1^), daily dry season feed intake (kg TLU^-1^ d^-1^) is the daily intake level during the rainy season, and daily dry season feed intake (kg d^-1^) is the daily feed intake during the dry season. The intake levels estimated from this equation were then aggregated across LPS based on the GPS coordinates of the households, to derive average annual feed intakes representative of MRT and MRH systems for the 6 feeds included in the model. The resulting values, which are the annualized feed on offer for the MRT and MRH systems in the model simulations, are shown in Table S5 (the ranges includes the ranges between MRT and MRH systems).

*Seasonal variation in feed supply*

From the annual feed intake as described above, the monthly feed availability was then determined taking into account practices influencing seasonal availability of feed (Table S3). This framework takes into account the seasonality of feed production based on the monthly biomass availability from each feed category, accounting for grazing practices, harvest dates, and rationing practices. The seasonal variation in yield of forages were obtained from ^[93]^. Crop stovers are available during the dry season, through either grazing on crop land or from harvested and rationed crops on farm ^[12]^. Concentrate feeds acquired off farm are the only feeds not affected by seasonality (i.e. they are available year-round). However, their feeding to cows is specified in LivSim in relation to the production stage of the animal (lactating, dry, gestating) as described in the scenarios section of the text. The quality parameters for each of the feed types for dry and rainy seasons were specified based on literature and FAO databases (Table S4).

Table S3: Conditions affecting seasonal availability of feeds

| Feed type | Seasonality conditions |
| --- | --- |
| Grass | Can be harvested or grazed year-round. |
| Pasture |  |
| Napier | Can be harvested or grazed year-round. |
| Maize stover | Available during dry season, by either grazing cattle on croplands (after harvest) or harvesting and providing to cattle *via* cut-and-carry. |
| Sunflower cake, maize bran | Available year round (purchased from the market). Can be feed to cows according to production cycle: early lactation (first 150 days), late lactation, gestation. |

Table S4: Nutrient properties of feed types by season

|  | Dry matter  (g kg^-1^) | | Dry matter digestibility  (%) | | Metabolisable energy  (MJ kg DM^-1^) | | Crude protein  (g kg^-1^) | | Acid  detergent  fibre  (g kg^-1^) | | Neutral  detergent fibre  (g kg^-1^) | |
| --- | --- | --- | --- | --- | --- | --- | --- | --- | --- | --- | --- | --- |
|  | Dry | Wet | Dry | Wet | Dry | Wet | Dry | Wet | Dry | Wet | Dry | Wet |
| Native grasslands ^1,2,a^ | 850 | 155 | 41.5 | 55.3 | 5.8 | 7.7 | 59 | 78 | 477 | 450 | 767 | 738 |
| Managed  Pastures^1,3,b^ | 850 | 155 | 45.0 | 65.0 | 6.5 | 8.6 | 63 | 94 | 477 | 423 | 800 | 725 |
| Napier grass^1^ | 893 | 179 | 53.7 | 61.4 | 6.2 | 8.2 | 97 | 103 | 419 | 425 | 711 | 715 |
| Maize stover^1^ | 928 | 296 | 46.8 | 56.7 | 6.9 | 8.4 | 39 | 68 | 396 | 496 | 699 | 750 |
| Maize stover urea molasses treated^1,4^ | 928 | -- | 46.8 | -- | 6.9 | -- | 100 | -- | 501 | -- | 800 | -- |
| Maize bran^1^ | 887 | | 72.4 | | 11.0 | | 119 | | 145 | | 442 | |
| Sunflower cake^1^ | 890 | | 61.1 | | 9.1 | | 324 | | 320 | | 450 | |

# Sources :

^1^ [94]

^2^ [95]

^3^ [96]

^4^ [97]

^5^ [62]

Table S5: Range of values provided to LivSim as feed on offer across production systems (MRT and MRH) for baseline simulations.

| Cohort | % of Dry matter | | | | | | Annual feed on offer (kg DM head^-1^) |
| --- | --- | --- | --- | --- | --- | --- | --- |
|  | Native grasses | Managed Pasture | Maize  stover | Napier  grass | Maize  bran | Sunflower  cake |  |
|  | Local | | | | | | |
| Cows | 47-50 | 0-3 | 31-36 | 1-2 | 12-14 | 0-2 | 2811$\pm$562 |
| Heifers | 48-54 | 5-6 | 31-36 | 0-1 | 8-10 | 0 | 2555$\pm$511 |
| Female  calves | 48-54 | 5-6 | 31-36 | 0-1 | 8-10 | 0 | 2190$\pm$438 |
| Bulls | 48-54 | 5-6 | 31-36 | 0-1 | 8-10 | 0 | 8500$\pm$1700 |
| Juvenile  males | 48-54 | 5-6 | 31-36 | 0-1 | 8-10 | 0 | 8000$\pm$1600 |
| Male  calves | 48-54 | 5-6 | 31-36 | 0-1 | 8-10 | 0 | 2190$\pm$438 |
|  | Improved | | | | | | |
| Cows | 6-7 | 19-21 | 12-17 | 32-35 | 10-12 | 8-13 | 3614$\pm$723 |
| Heifers | 16-17 | 24-25 | 12-17 | 32-35 | 10-11 | 0 | 3541$\pm$708 |
| Female  calves | 16-17 | 24-25 | 12-17 | 32-35 | 10-11 | 0 | 2519$\pm$504 |
| Bulls | 16-17 | 24-25 | 12-17 | 32-35 | 10-11 | 0 | 3650$\pm$730 |
| Juvenile  males | 16-17 | 24-25 | 12-17 | 32-35 | 10-11 | 0 | 3577$\pm$715 |
| Male  calves | 16-17 | 24-25 | 12-17 | 32-35 | 10-11 | 0 | 2519$\pm$504 |

Notes: Standard errors reported for dry matter intake represent range of error used in uncertainty analysis

**2. Calculation of direct greenhouse gas emissions sources**

Based on the feed intake from feed on offer as calculated from LivSim, emissions from enteric fermentation, manure, and managed soils were calculated according to the updated IPCC (2019) methodology ^[98]^, however for consistency this paper will still refer to IPCC (2006). The managed soils included in this assessment extend to the land categories included as part of the dairy land footprint as described in Table 1 of the text. All values were first calculated as an annual per livestock unit, expressed as CO_2_ equivalents, and then aggregated to calculate GHG emissions for each production system, taking into account the number of cattle in each production system (as described above). Within the study region, the predominant manure management system is solid storage ^[99]^ however there is significant variation in the percentage of manure that is managed versus excreted on pasture. In the present study manure emissions from CH_4_ includes manure that is managed and excreted on pasture. Manure N_2_O includes only managed manure, and N_2_O emissions from manure applied or excreted on soils is included as N_2_O emissions from crop and grassland soils, according to IPCC (2006) chapter on N_2_O emissions from managed soils.

Methane from enteric fermentation was estimated as a percentage of gross energy intake per animal using the following equation from ^[100]^ :

Y_m_ = 3.5 + 0.243 x DMI + 0.0059 x ADF + 0.057 x DMD (2)

Where Y_m_ is the methane conversion factor (% of gross energy converted to CH_4_), DMI is dry matter intake (kg head^-1^ day^-1^), ADF is intake of acid detergent fibre (g kg^-1^ DM), and DMD is dry matter digestibility (g kg^-1^ DM). Manure CH_4_ was estimated based on volatile solids, methane producing capacity (B_o_), and the methane conversion factor (MCF) using IPCC (2006) equations 10.23 and 10.24. The methane producing capacity took a value of 0.13 m^3^ CH_4_ kg VS^-1^, which is the IPCC default value for the African continent (IPCC 2006). The MCF was calculated as weighted average for each livestock production system and breed of cattle based on the default MCF values for solid storage and pasture (Table SM 5).

Manure N_2_O was calculated as the sum of direct N_2_O from nitrification and denitrification of manure nitrogen, and indirect N_2_O from volatilization and leaching of N in storage. Nitrogen excretion quantified by LivSim was used to calculate direct and indirect N_2_O emissions based on equations 10.25, 10.26 and 10.27 from IPCC (2006). Again, IPCC (2006) default emission factors for solid storage systems and excretion on pasture were used.

The fraction of manure N available for soil application was based on the fraction stored minus the amount lost from directly and indirectly through volatilization and leaching. This along with the manure N excreted on grasslands was then used as an N input into soils, which was then used in accordance with the IPCC (2006) framework for soil N_2_O emissions, which includes N_2_O emissions from manure, inorganic fertilizer and residue N (equations 11.1, 11.9, 11.10, and 11.11). For manure excreted on grasslands, a Tier 2 emission factor was used (taking a value of 0.00105) based on field experimental studies in the region ^[101]^. Application rates of N fertilizer took values of 20 kg N ha^-1^ yr^-1^ for maize and sunflower, and 10 kg N ha^-1^ yr^-1^ for food crops, representing typically observed application rates for the southern highlands region of Tanzania ^[102] [103]^. It was assumed no fertilizer was applied on forage crops or grasslands. N from crop residues and forage/pasture renewal were calculated for each feed with values taken from table 11.2. For food crops the fraction removed was set at 0.5. Mass based allocation factors on N_2_O emissions from cropland dedicated to stover and concentrate production in order to distinguish between the fraction consumed as feed and co-products. These allocation factors were based on the ratio of feed biomass to total biomass yield (Table 1 in main text). The resulting (baseline) N_2_O emissions for the three cropland types and two forages (before allocation) are shown in Table 1 of the text. All the emission factors used in the study and their sources are shown in Table S5.

Emissions associated with the production of inputs produced upstream from the farm were included in the model as ‘Energy use CO_2_’. These sources extend to the emissions associated with processing and transporting concentrate feeds, and for manufacturing fertilizer. The predominant concentrate feeds used in the southern highlands, maize bran and sunflower cake, are grown and processed domestically ^[37] [38]^. The emissions associated with transportation were based on an average travel distance from the point of feed processing to the farm of 200 km. The coefficients from fossil energy use were based on [104]. The energy requirements for feed processing took values of 186 MJ of electricity and 188 MJ of gas per 1,000 kg of feed DM. For this production energy requirement and an average travel distance of 200 km, an embodied feed emission factor of 0.0786 kg CO_2_ eq kg compound feed^-1^ was derived. CO_2_ emissions from manufacturing and transport of fertilizers were based on the fertilizer use values listed per feed category as listed above, and using an embodied emission factor of 5.66 kg CO_2_ kg N^-1^ ^[105]^. The total value for ‘Energy use CO_2_’ emissions were thus based on the sum of emissions from feed processing and transport and manufacturing of fertilizer.

Table S6: Emission factors used in attributional life cycle assessment of dairy sector

| Emission factor | Value | Source |
| --- | --- | --- |
| Y_m_ | Estimated as in Jaurena et al. (2016) | [100] |
| ^a^ MCF | 0.015 (pasture)  0.04 (solid storage) | [30] |
| ^a^ EF_3_ storage (direct manure N_2_O) | 0.005 | [30] |
| ^a^ EF_3_ pasture (direct manure N_2_O) | 0.00105 | [101] |
| ^a^ EF_4_ (indirect manure N_2_O) | 0.01 | [30] |
| ^a^ EF_5_ (indirect manure N_2_O) | 0.0075 | [30] |
| ^a^ Fraction N volatilized -- pasture | 0.2 | [30] |
| ^a^ Fraction N leached -- pasture | 0.3 | [30] |
| ^a^ Fraction N volatilized – solid storage | 0.3 | [30] |
| ^a^ Fraction N leached – solid storage | 0.4 | [30] |
| EF_1_ (soil N inputs) | 0.0105 (inorganic N), 0.01 (organic N) | [68] [30] |
| EF_5_ (leaching and runoff) | 0.0075 | [30] |
| Fraction gas volatilized (organic N) | 0.1 | [30] |
| Fraction gas volatilized (synthetic N) | 0.2 | [30] |
| Fraction lost manure management | 0.4 | [30] |

^a^ Specified in the model for each production system as a weighted average based on the fraction of manure excreted on pasture vs. managed, as estimated from GLS (2019)

**3. Spatial estimation of grasslands availability and utilization**

The conversion of woody native ecosystems occurs in the model when the requirement for grasslands exceeds the availability of feed per spatial unit (100 km^2^). The availability of grasslands and percentage utilized for grazing and cut and carry feeding were estimated based on the land cover data ^[58]^,the cattle population densities ^[70]^ ,and the parameters specified to reflect productivity and efficiency of grazing/harvesting of grassland species included in the model. The feed categories described in the body of the paper, which were included in this framework, were all feed categories that are not included under the crop category for the ^[58]^ data. This includes Napier grass, managed pasture, and native grasslands. The extent of grassland utilization was calculated with the following equation:

Grassland utilization = $\frac{Cattle density \times Grass consumption \times Use efficiency}{Grassland yield}$ (3)

Where grassland utilization (km^2^) is the extent of grasslands per spatial unit being utilized for ruminants, cattle density (head km^-2^) is based on ^[70]^, grass consumption (Mg DM head^-1^ yr^-1^) is the grass consumption per animal as specified above, utilization efficiency is the fraction of grass available that is harvested or consumed by grazing cattle (Table 1 of text), and grassland yield is the yield of grassland (Mg DM ha^-1^ yr^-1^) (Table 1 of main text).

In the final year of the model simulation period (2030) the grassland available for use by the dairy sector was equal to grassland area in the base year (2020) minus the expected expansion from non-dairy sector sources. These sources include cropland as an aggregate, and the grassland occupied for grazing by beef cattle. Cropland expansion was calculated based on the crop land area in the base year ^[58]^ and the annual growth rate as calculated from FAO data ^[38].^ The growth rate in land needed for beef cattle grass consumption was calculated based on the beef cattle population and the land requirement for their grass consumption, which was calculated from ^[43]^.

**4. Modelling yield gains and nitrous oxide emissions from N-fertilizer**

The results of the calculations used to simulate yield gains and N_2_O emissions are reported here. These simulations only extend to maize and sunflower used for producing concentrate feeds (maize for producing bran and sunflower for producing cake), reasoning that commercial oriented producers would have adequate technical and managerial capacities to efficiently increase fertilizer use, while the majority of financial and labour constrained smallholder (dairy) producers have low technical capacity to adequately apply fertilizers [106]. Moreover, developing the commercial feed production and processing industries for maize and sunflower are part of the broader component for developing Tanzania’s dairy industry [65].

The yields of maize and sunflower were revised from their regional average values of 1.46 (maize) and 1.03 (sunflower) Mg ha^-1^ yr^-1^ ^[38]^ upwards by 50% of the yield gap, thus taking values of 3.71 and 2.03 Mg ha^-1^ yr^-1^. The N-fertilizer application rates in the *baseline* yield scenario take values of 20 kg N ha^-1^ yr^-1^ and in the 50% yield gap scenario these are increased to 161.0 and 69.5 kg N ha^-1^ yr^-1^. In *baseline* yield the N_2_O fluxes for maize and sunflower (calculated based on IPCC methodology in SM 2) are estimated at 1.03 (maize) and 0.9 (sunflower) kg N_2_O ha^-1^ yr^-1^. In *50% yield gap* these values increase to 5.68 (maize) and 2.9 (sunflower) kg N_2_O ha^-1^ yr^-1^. In the *baseline* scenario the yield scaled N_2_O emissions thus take values of (maize) 1.03 kg N_2_O ha^-1^ yr^-1^ / 3.71 Mg ha^-1^ yr^-1^ = 0.28 kg N_2_O Mg^-1^ and (sunflower) 0.9 kg N_2_O ha^-1^ yr^-1^ / 1.03 Mg ha^-1^ yr^-1^ = 0.87 kg N_2_O Mg^-1^. In the *50% yield gap* scenario the yield scaled N_2_O emissions take values of (maize) 5.67 kg N_2_O ha^-1^ yr^-1^ / 3.71 Mg ha^-1^ yr^-1^ = 1.53 kg N_2_O Mg^-1^ and (sunflower) 2.9 kg N_2_O ha^-1^ yr^-1^ / 2.03 Mg ha^-1^ yr^-1^ = 1.43 kg N_2_O Mg^-1^. Thus, while greater N application rates up to 161.0 and 69.5 kg N ha^-1^ yr^-1^ for maize and sunflower, respectively, increase yields (and hence reduce the dairy land footprint), total N_2_O emissions per hectare and per unit yield increase.

**5.** Sources of uncertainty

Table S7: Sources of uncertainty

| Variable used in model | Relative standard error |
| --- | --- |
| Grassland yields | +/- 20 |
| Maize yield | +/- 20 |
| Sunflower yield | +/- 20 |
| Cattle populations | +/-20 |
| Feed intake per tropical livestock unit | +/-25 |
| Y_m_ | +/- 10 |
| Bo | +/- 30 |
| MCF | +/- 20 |
| EF_1_ (soil N inputs) | +/- 66 |
| EF_3_ storage (direct manure N_2_O) | +/- 30 |
| EF_3_ pasture (direct manure N_2_O) | +/- 7 |
| EF_4_ (indirect manure N_2_O) | +/- 30 |
| EF_5_ (indirect manure N_2_O) | +/- 30 |
| Fraction N volatilized -- pasture | +/- 7 |
| Fraction N leached -- pasture | +/- 7 |
| Fraction N volatilized -- storage | +/- 7 |
| Fraction N leached -- storage | +/- 7 |
| EF_4_ (atmospheric deposition) | +/- 30 |
| EF_5_ (leaching and runoff) | +/- 30 |
| Fraction gas volatilized (organic N) | +/- 30 |
| Fraction gas volatilized (synthetic N) | +/- 30 |
| Fraction lost manure management | +/- 30 |
| C stock density croplands | +/- 20 |
| C stock density grasslands | +/- 20 |
| C stock density native ecosystems | +/- 20 |
| Embodied feed and fertilizer footprints | +/- 30 |

**6.** Dry season milk yield and nutrient scarcity by feed scenario

Table S8: Yield and dry season nutrient deficits for cows across feeding scenarios

| Scenario | Mixed rainfed tropical | | | Mixed rainfed humid | | |
| --- | --- | --- | --- | --- | --- | --- |
|  | Local cows | | | | | |
|  | Milk yield (kg hd^-1^ yr^-1^) | Metabolisable energy deficit (MJ d^-1^) | Metabolisable protein deficit  (g d^-1^) | Milk yield  (kg hd^-1^ yr^-1^) | Metabolisable energy deficit  (MJ d^-1^) | Metabolisable protein deficit  (g d^-1^) |
| *Base* | 358 | 14 | 19 | 331 | 15 | 6 |
| *L-Cn* | 424 | 13 | 0 | 377 | 16 | 3 |
| *L-Fo* | 472 | 12 | 25 | 425 | 13 | 21 |
| *L-CnFo* | 507 | 11 | 0 | 466 | 9 | 2 |
| *L-Co* | (infeasible; results in mortality due to undernutrition in non-lactating periods) | | | | | |
| *L-FoCo* | 437 | 12 | 10 | 47 | 7 | 0 |
| *L-CnFoCo* | 528 | 13 | 0 | 501 | 12 | 2 |
|  | Improved cows | | | | | |
| *Base* | 932 | 14 | 19 | 875 | 15 | 6 |
| *I-Cn* | 991 | 13 | 0 | 915 | 16 | 3 |
| *I-Fo* | 1207 | 9 | 7 | 1035 | 9 | 4 |
| *I-CnFo* | 1264 | 12 | 0 | 1059 | 9 | 2 |
| *I-Co* | 1049 | 7 | 0 | 12 | 32 | 0 |
| *I-FoCo* | 1458 | 6 | 3 | 1335 | 12 | 3 |
| *I-CnFoCo* | 1492 | 13 | 0 | 1355 | 12 | 2 |
